# Biofabricated macrophage and fibroblast membranes synergistically promote skin wound healing

**DOI:** 10.1002/btm2.10344

**Published:** 2022-06-03

**Authors:** Dongqing Wang, Heying Chen, Li Lei, Jun Chen, Jimin Gao, Jiahe Liu, Qianyin Li, Yajun Xie, Yi Hu, Yilu Ni

**Affiliations:** ^1^ The M.O.E. Key Laboratory of Laboratory Medical Diagnostics, The College of Laboratory Medicine Chongqing Medical University Yuzhong District Chongqing China; ^2^ CAS Key Laboratory for Biomedical Effects of Nanomaterials and Nanosafety Institute of High Energy Physics and University of Chinese Academy of Sciences (UCAS), Chinese Academy of Sciences (CAS) Beijing China; ^3^ Zhejiang Provincial Key Laboratory for Technology & Application of Model Organisms, School of Laboratory Medicine and Life Science Wenzhou Medical University, University Town Wenzhou Zhejiang China

**Keywords:** cytomembrane, fibroblast, macrophage, wound healing

## Abstract

Effective skin wound healing is a complex process involving anti‐inflammation, fibrosis, matrix reconstruction, and angiogenesis. This work aimed to integrate the macrophage‐mediated anti‐inflammation and fibroblast‐assisted matrix reconstruction for enhanced skin wound healing. Herein, we utilized the cytomembranes derived from repolarized M2 macrophages and fibroblasts to prepare the natural biologics. Results showed that the inflammatory M1 macrophages were repolarized to M2 phenotype by the M2 macrophage cytomembranes. As a consequence, the cytomembranes of M2 macrophage could facilitate the wound closure in mice. Furthermore, the addition of fibroblast membranes to the macrophage cytomembranes contributed to a better matrix reconstruction, neovascularization and angiogenesis. Next, we used a transforming growth factor‐β (TGF‐β) inhibitor to attenuate cutaneous scar formation. Therefore, our modality could promote skin wound healing and effectively suppress scar formation in the preclinical murine skin wounds. The cytomembrane biologics might provide a biocompatible and versatile tool for wound healing.

## 1 INTRODUCTION

Skin wound healing is a complex physiological process including four major phases: hemostasis, inflammation, proliferation, and tissue remodeling.^1^ In the dynamic healing process, hemostasis first induces the blood coagulation to cause instant blood clotting.^2^ And then, the inflammation process follows as the decisive phase to combat against bacterial infection and to eliminate damaged cells.^3^ During this period, macrophage plays the most important role to present diverse biofunctions such as phagocytosis, promoting inflammation, and producing various cytokines that stimulate new capillary growth, collagen synthesis, and tissue fibrosis.^4^, ^5^, ^6^ Once the wound is cleaned up by inflammation, the next phase defined as proliferation is demanded for new generated tissues to fill and cover the wound surface.^7^ As the key reparative cell, fibroblast closely correlates with tissue regeneration due to its immigration along the fibrin network.^8^, ^9^, ^10^ It initiates re‐epithelization from the wound edges by secreting fibronectin and collagen, which contributes to matrix reconstruction.^11^ Meanwhile, neovascularization and angiogenesis get activated, serving for the wound closure between tissue gaps.^12^ Eventually, the regeneration gradually ceases in the following remodeling phase, new generated tissues are reorganized.^13^ To be noted, the M2 macrophage also play another important role in directing fibroblasts to express genes and secrete proteins, thus generating the M2 macrophage‐fibroblast crosstalk to promote extracellular matrix (ECM) deposition.^14^

Until now, numerous studies have been focused on the modulation of cellular behaviors of both macrophage and fibroblast, chasing positive resolution of inflammation and supportive cell proliferation.^15^, ^16^, ^17^ Very recently, membrane has been developed as an efficient tool kit in biomaterial research.^18^, ^19^, ^20^ The allogenic or autologous provenance of cytomembrane effectuates informative communication being conveyed directly to targeted cells or tissues with high efficiency and excellent biocompatibility.^21^, ^22^ In most cases, cytomembranes are used as decoys or carriers to deliver drugs or nanoparticles, preventing them from being captured and cleared away by host mononuclear phagocyte system (MPS).^23^, ^24^, ^25^ Moreover, specific biological molecules (e.g., growth factors, and signal molecules) are expressed on the surface of cytomembranes or even their fragments, endowing them with peculiar biofunctions.^26^, ^27^ However, most of the cytomembrane strategies are involved with chemical materials or synthetic drugs, the biosecurity problems like chemical toxicity or long‐term retention are of great concern in clinical trial.^28^, ^29^ Previously, we have manufactured a tumor vaccine based on fused cytomembrane (derived from macrophages) to induce robust immune responses and prevent tumor recurrence.^30^ Thus, we intend to take further advantages of the bio‐affinitive and co‐stimulatory merits in cytomembrane, proposing a chemical materials‐, exogenous antigens‐ and adoptive cells‐free modality to provide a biosafe solution for skin wound repair.

**SCHEME 1:**
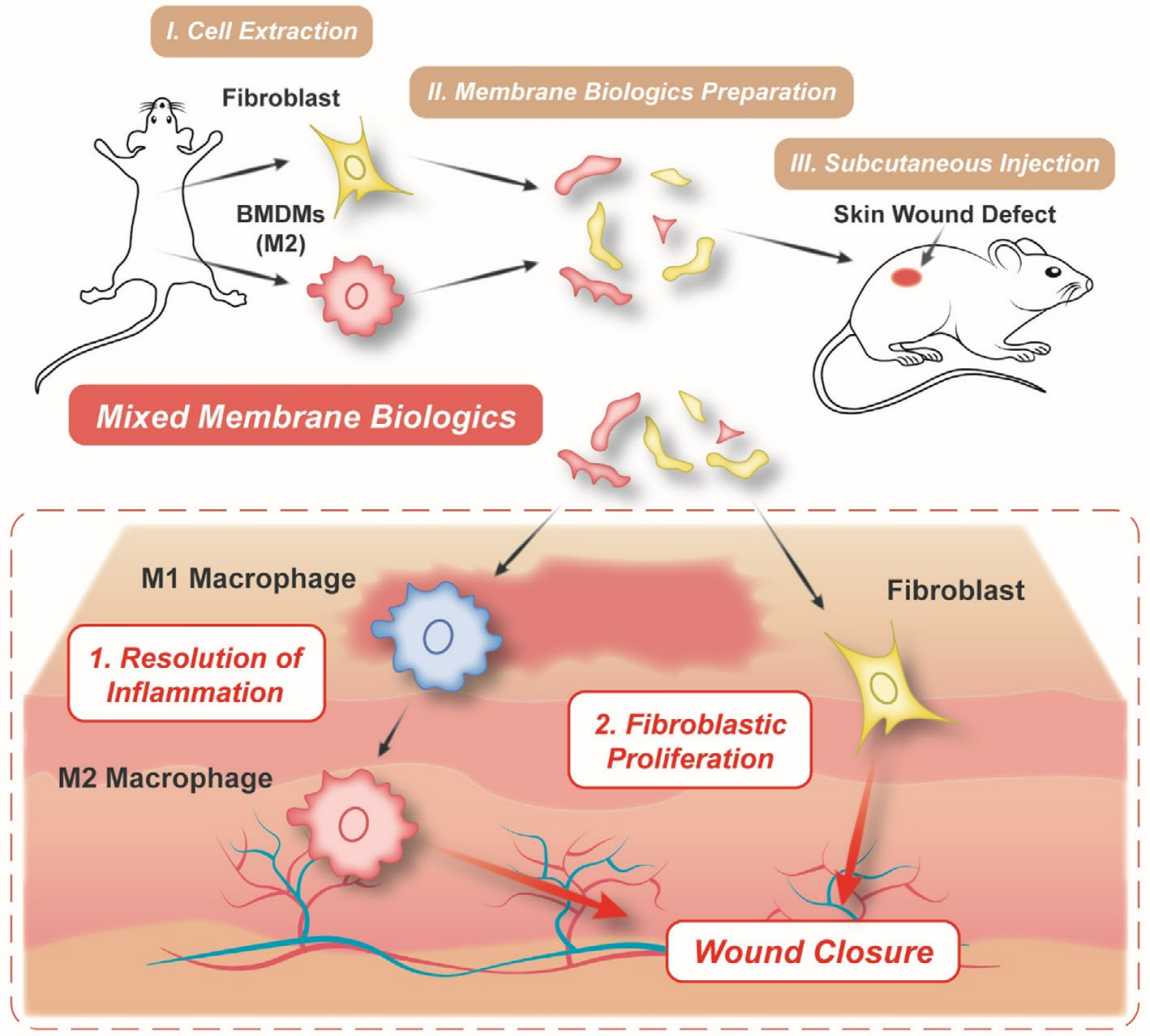
Schematic illustration of the mixed membranes (MM) biologics that promote skin wound healing.

Herein, allogenic macrophages and fibroblasts were first obtained from the bone marrow and the skin of mice, respectively (Scheme 1). The macrophages were induced into M2‐like phenotype in conditioned culture medium. The cytomembranes of M2 macrophages and fibroblasts were then extracted and mixed into the suspended biologics (named as MM). This cytomembrane biologics can efficiently reverse over‐inflammatory M1 macrophages to M2 phenotype due to the inclusive anti‐inflammatory macrophage membranes, accelerating the “inflammation” phase of the wound healing cascade. Next, significant angiogenesis and re‐epithelization were both activated and restored by the fibroblast membranes in the MM biologics, expediting the “proliferation phase.” These positive bioreactions quickly built the basis for the generation of new matrix, serving for the full‐thickness wound closure. Finally, in order to reduce excessive collagen deposition and to avoid disfiguring scars, we intentionally intervened the scarring process by injecting TGF‐β inhibitor in the late stage to develop scarless healing. In all, this cytomembrane‐based strategy modulated two dominating cells (i.e., macrophage and fibroblast) in the inflammation and proliferation phases of wound healing. This strategy may provide new insight for the safe and efficient skin wound repair and shed some light on the future of nature‐derived biomaterials.

## 2 RESULTS

### 2.1 Extraction and characterization of allogenic macrophages and fibroblasts

**FIGURE 1:**
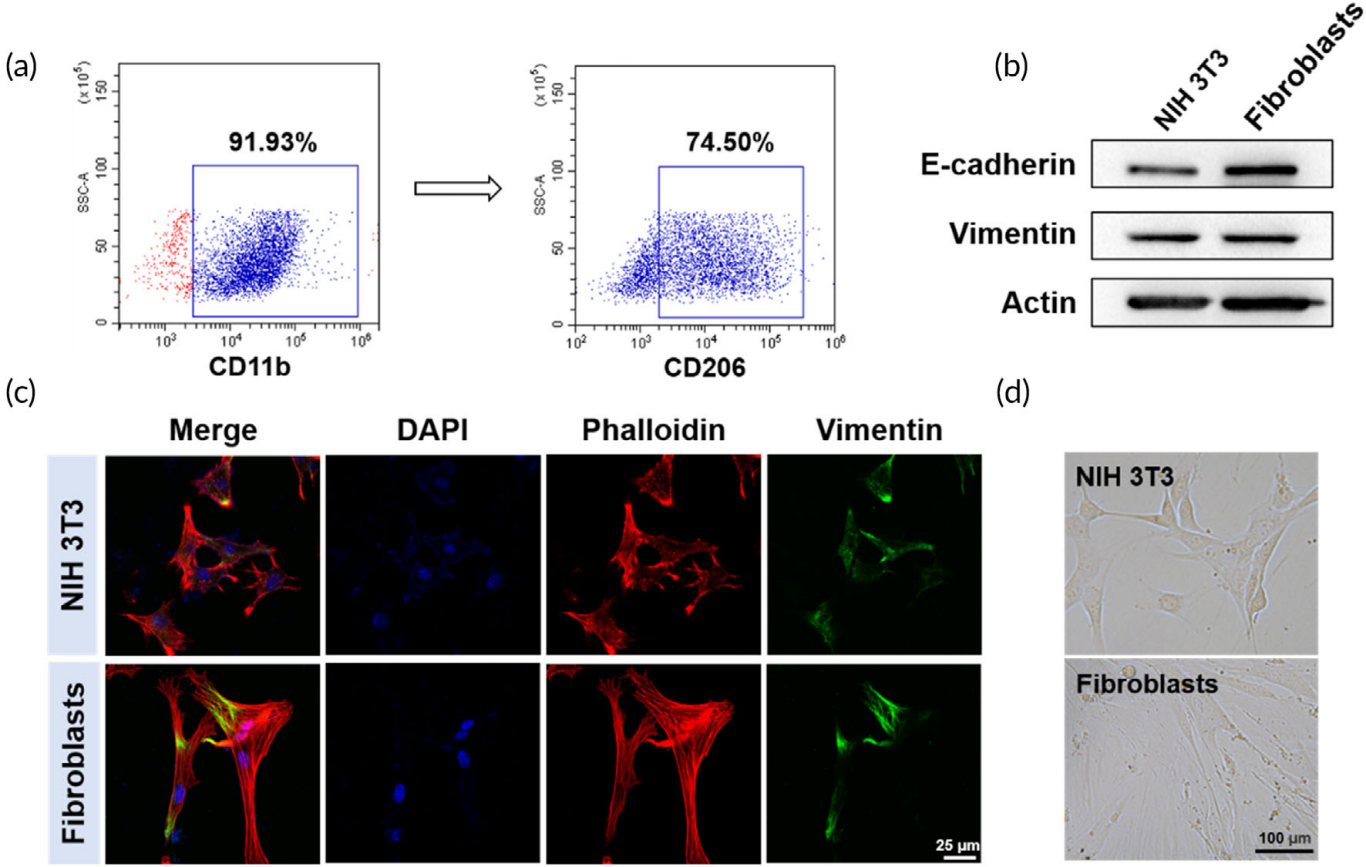
Characterization of endogenous fibroblasts and M2 macrophages derived from bone marrow‐derived macrophages (BMDMs). (a) Flow cytometry analysis of M2 macrophages (CD11b^+^ CD206^+^) derived from BMDMs. (b) The expression of E‐cadherin and Vimentin in extracted fibroblasts and NIH 3T3 cells. (c) The fluorescent images of extracted fibroblasts and NIH 3 T3 cells. Cell nucleus were stained with Hoechst 33342 in blue, Phalloidin and Vimentin were marked in red and green, respectively. (d) The morphology of extracted fibroblasts and NIH 3T3 cells

The bone marrow‐derived macrophages (CD11b^+^) were successfully obtained from BALB/c mice and polarized into M2‐phenotype (CD206^+^) in conditioned culture medium (Figure 1a). Likewise, fibroblasts were extracted from the skin of the identical BALB/c mice. And the extracted fibroblasts showed the same expression level as another fibroblast cell line, namely, NIH 3T3, in the marker proteins such as E‐cadherin and Vimentin demonstrated by Western Blot (Figure 1b). In addition, these two fibroblast cell lines showed great resemblance in morphology, the marker protein Vimentin was fluorescently visualized (Figure 1c). Under microscope, the extracted fibroblasts appeared with a more spindle shape than NIH 3T3 (Figure 1d), which was completely rational considering its progenitor source from the skin.

### 2.2 The MM biologics regulate macrophages' polarity and prevent redundant inflammation

**FIGURE 2:**
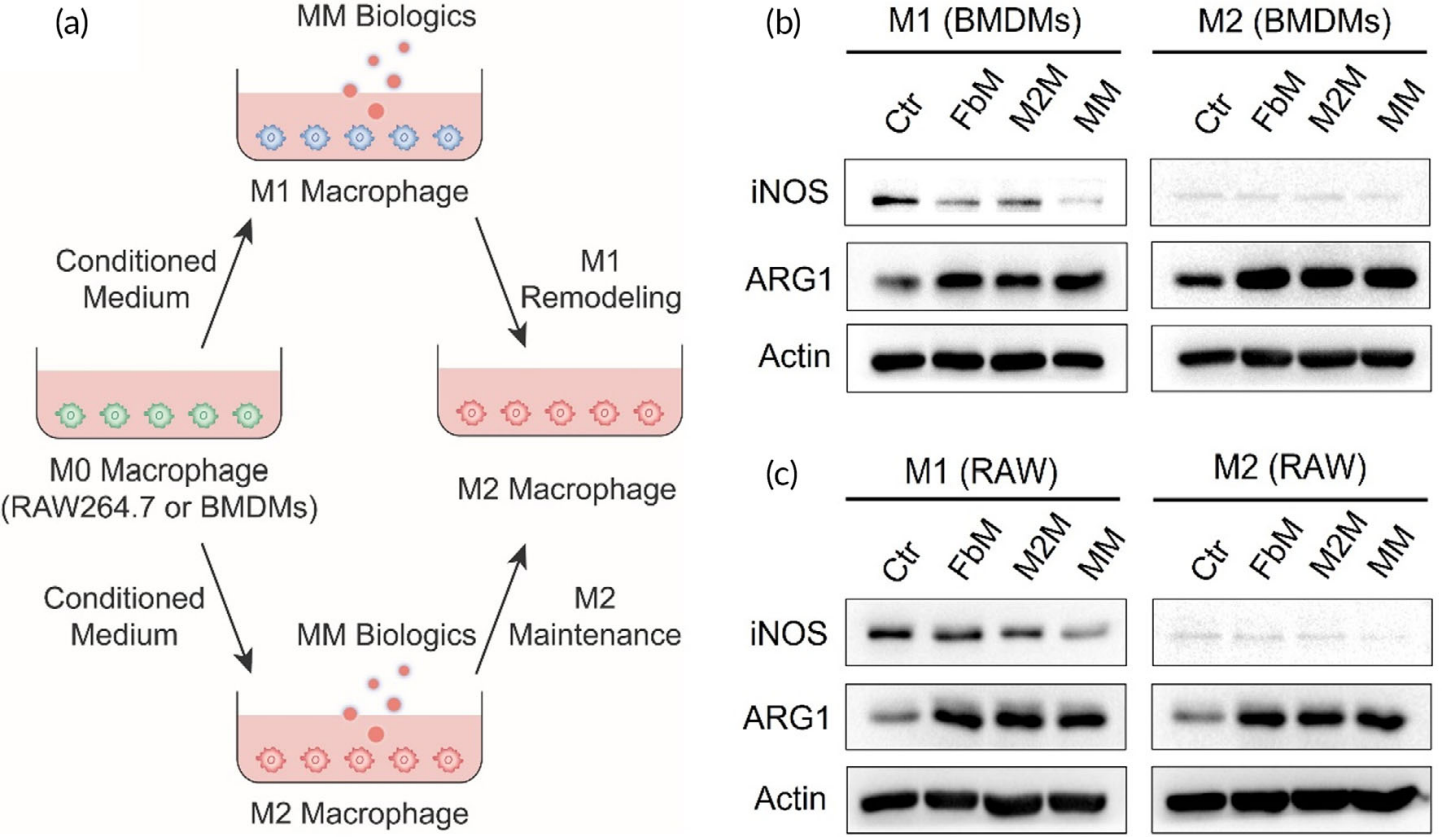
The mixed membranes (MM) biologics remodel M1 macrophages toward M2 phenotype. (a) Schematic diagram of experiment setup. (b) Expression of iNOS and ARG1 in M1 and M2 macrophages derived from bone marrow‐derived macrophages (BMDMs) or (c) RAW264.7 cells

Next, we obtained the cytomembranes of bone marrow‐derived macrophages (BMDMs) induced M2 macrophages and skin‐derived fibroblasts, these cytomembranes were mixed into membrane biologics (named MM) for further use (Scheme 1). Since the MM biologics contained the membrane fragments of M2 macrophages, we expected it to reprogram endogenous macrophages toward M2 phenotype for the resolution of inflammation. Thus, the polarity of macrophages in different subtypes was surveyed under membrane treatment. First, the extracted BMDMs or RAW264.7 cells were induced into M1 or M2‐like phenotype (Figure 2a), these two subtypes of macrophages were both treated (untreated as Ctr group) with fibroblast membranes (FbM group), M2 macrophage membranes (M2M group), or mixed membranes (MM group), respectively. As shown in Figure 2b, M1 macrophages (marked by iNOS) were polarized toward M2‐like phenotype (marked by ARG1) by MM, and the polarity of M2 macrophages was maintained. Moreover, to prove that the membrane biologics were broad‐spectrum in polarity regulation, another lab model macrophage, that is, RAW264.7 cell was also evaluated under the impact of cytomembranes. Likewise, the Western Blot showed similar results that MM induced all three types of RAW264.7‐derived macrophages toward M2‐phenotype (Figure 2c). These results verified that the membrane biologics could induce allogenic macrophages (BMDMs) and cell model macrophages (RAW264.7) into M2‐phenotype, regardless of the polarity they already presented.

### 2.3 The MM biologics promote migration, proliferation, and activation of fibroblasts

**FIGURE 3:**
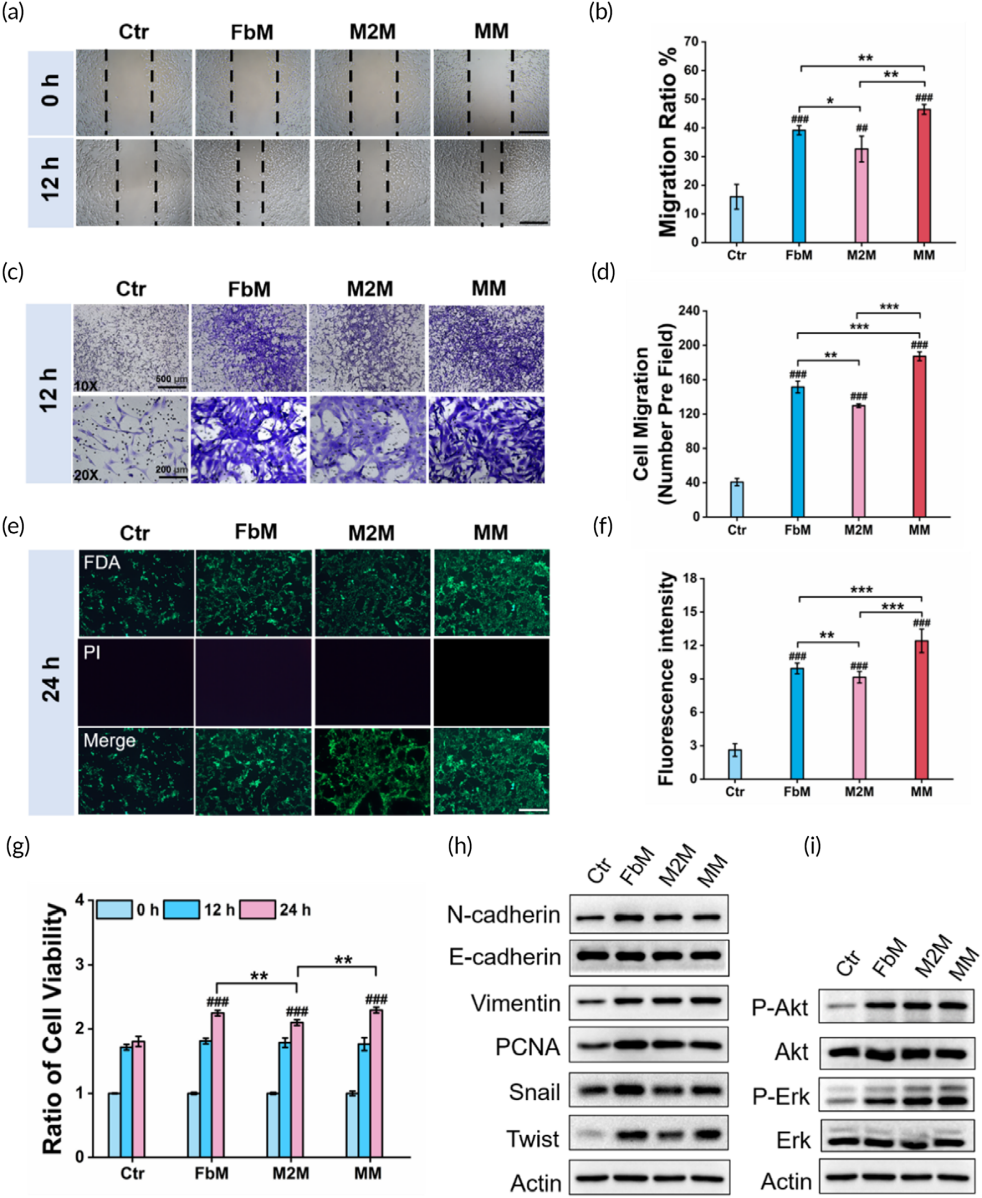
The mixed membranes (MM) biologics promote fibroblastic migration, proliferation, and activation. (a) Representative optical images and (b) the migration ratio of NIH 3T3 cells. Scale bar = 200 μm. (c) Representative images and (d) the invasion ratio of NIH 3T3 cells. Scale bar = 200 μm. (e) Representative fluorescent images of NIH 3T3 cells cultured for 24 h. Living/dead cells were stained with FDA (green) and PI (red), respectively. Scale bar = 200 μm. (f) The relative fluorescent intensity of living cells. (g) The relative cell viability of NIH 3T3 cells. (h) The expression of N‐cadherin, E‐cadherin, Vimentin, PCNA, Snail, and Twist in NIH 3T3 cells under different treatments. (i) Akt, Erk1/2 expression, and their phosphorylation levels in NIH 3T3 cells. “Ctr” represents untreated group. Data are presented as mean ± SD (*n* = 3). “#” is the intragroup comparison with “Ctr,” and “*” is the intergroup comparison. * or #, *p* < 0.05; ** or ##, *p* < 0.01; *** or ###, *p* < 0.001

We hypothesized that the MM biologics would promote the proliferation phase of wound healing by facilitating migration, proliferation, and activation of fibroblasts. To test this hypothesis, we first performed the scratch assay on the NIH 3T3 cell, which was certified as the cell model fibroblast,^31^ to determine the fibroblastic migration status. In Figure 3a,b, the NIH 3T3 cells showed the highest migration rate under the MM treatment, while the FbM and M2M were similarly less effective. Furthermore, the transwell chamber assay also validated that the MM biologics prompted more NIH 3T3 cells to migrate or invade (Figure 3c,d). In addition, the fluorescent living/dead cell images and CCK8 assay both confirmed that all the membranes accelerated the NIH 3T3 cells to proliferate (Figure 3e–g), in which the MM showed a significantly higher promotion. This phenomenon was also seen in two other cell lines of fibroblasts, that is, mouse embryonic fibroblast (MEF, Figure S1) and the skin‐derived fibroblasts (Figure S2), indicating the general effect of MM on fibroblastic proliferation. Next, Western Blot was performed on NIH 3T3 cells after 24 h of treatment with different membranes. In Figure 3h, the expression of proliferating cell nuclear antigen (PCNA) indicated the accelerated proliferation pattern of NIH 3T3 cells under the treatment of cytomembranes. Moreover, two important transcription factors (Snail and Twist) were activated. Besides, the phosphorylated Akt and Erk1/2 were also found to be upregulated by cytomembranes (Figure 3i). However, the loss of E‐cadherin, the increase of N‐cadherin or Vimentin was not evident. All these results seemingly implied to a strong effect of cytomembranes to induce fibroblasts express signaling factors that were critical for matrix construction or vascularization, among them the fibroblast membrane (FbM) showed the strongest effect. When the same experiments were performed in the extracted fibroblasts (Figures S3 and S4), similar expression pattern was observed, reconfirming the general influence of cytomembranes on fibroblasts with different progenitor sources.

### 2.4 The MM biologics accelerate wound healing in vivo

**FIGURE 4:**
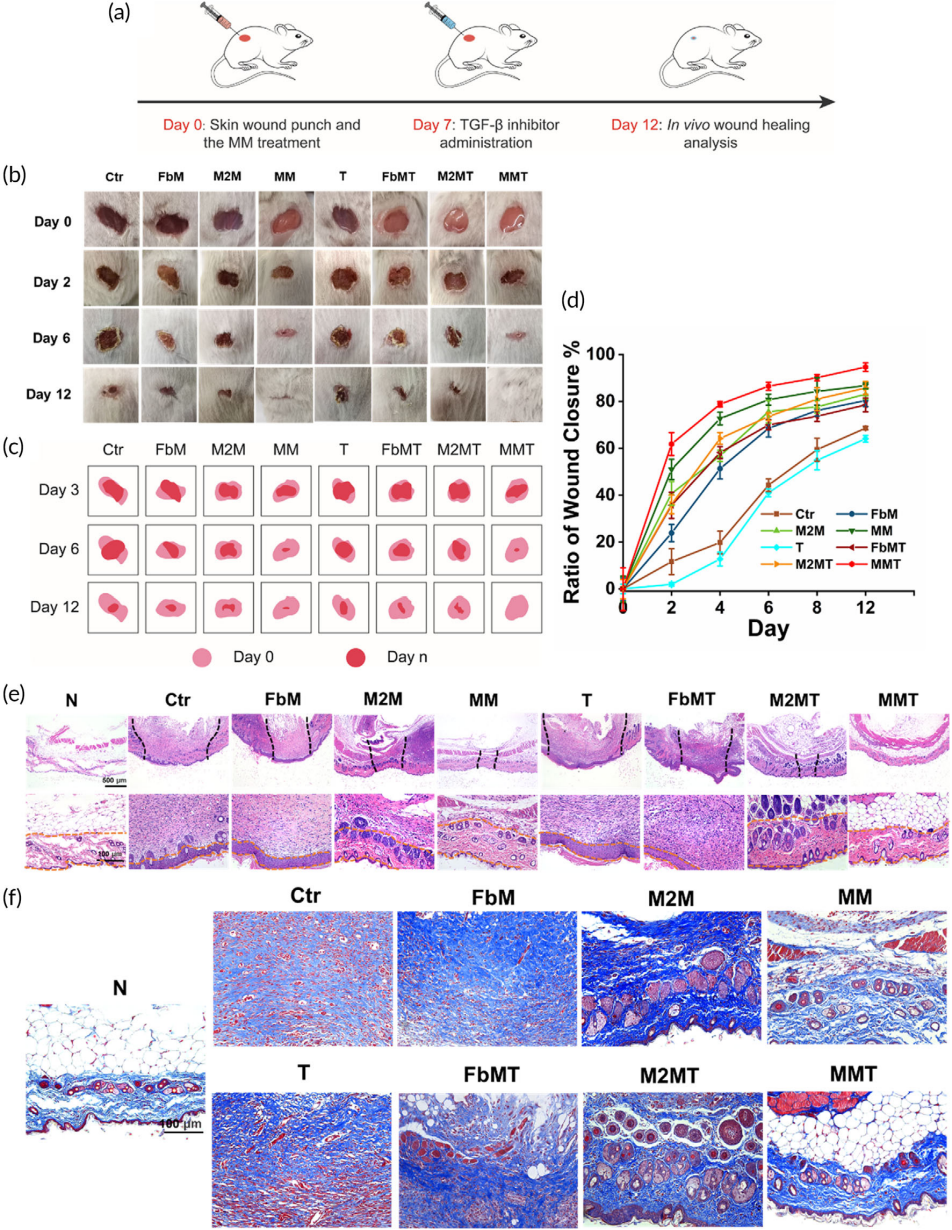
In vivo wound healing evaluation. (a) Treatment schedule. (b) The progression of the wound defect area. The wound diameter was 8 mm at Day 0. (c) Simulation diagram. (d) Quantitative evaluation of the wound closure rate. (e) The H&E and (f) Masson staining images of the wounds

To evaluate the in vivo healing effect of the MM biologics, the skin defect animal model was built in BALB/c mice. Figure 4a showed the treatment schedule of the skin wound. The mice were retrieved at specific time intervals and fluorescently imaged in Figure S5. The DiO‐stained MM biologics were injected around the wound edges and was degraded or cleared away within 4 days. In the photographs acquired with a digital camera (Figure 4b), simulation diagrams (Figure 4c) and quantitative ratio of wound closure results (Figure 4d) of the wounds in 12 days, the MM biologics showed the strongest effect of accelerating the wound healing process. The wounds treated by MM or M2M were healed significantly faster than the control (Ctr) and FbM‐treated groups in the first 4 days (Figure 4d). But the healing rate was slowed down in the M2M‐treated wounds in the next week, while the MM continued to promote wound closure. When the TGF‐β inhibitor was injected at Day 7, it did not show much favorable effect in the T group, which was not treated by any type of membranes before (Figure 4c). And also, the inhibitor failed to show preferable results in FbMT and M2MT groups comparing to FbM or M2M groups, respectively. In contrast, the MM's capacity to promote wound healing was further enhanced by the inhibitor, even succeeded to suppress scarring at the wound edges in the late stage (Figure 4b). To show the general effect of the MM biologics, the animal study was also performed in the KM mice model. Similar results were seen in Figures S6–S9, implying that the MM biologics could also promote heterogeneous wound healing.

In the H&E staining images illustrated in Figure 4e, the MM‐treated group had smaller wound area and thicker granulation tissue than the other groups, which was consistent with the quantitative analysis of the wound healing results in Figure 4b–d. In skin structure, collagen fibers are normally divided into types I and III. Type I collagen is the main component of skin tissue, while type III is the main component of reticular fibers. The normal ratio of collagen I and III maintains skin tissue structure. The results of Masson staining (Figures 4f and S10) showed that the collagen arrangement in the MMT group was more regular. Collagen fiber alignment was improved in the MMT group compared to the control group and was similar to that of the normal skin,^32^, ^33^ suggesting the positive effect of the MMT treatment on ECM deposition and collagen alignment. Importantly, the type I/III collagen ratio in the MMT group was more similar to the normal skin compared with the other groups (Figure S10). The results indicated that the combination of cell membrane and TGF‐β inhibitor promoted the wound healing.

**FIGURE 5:**
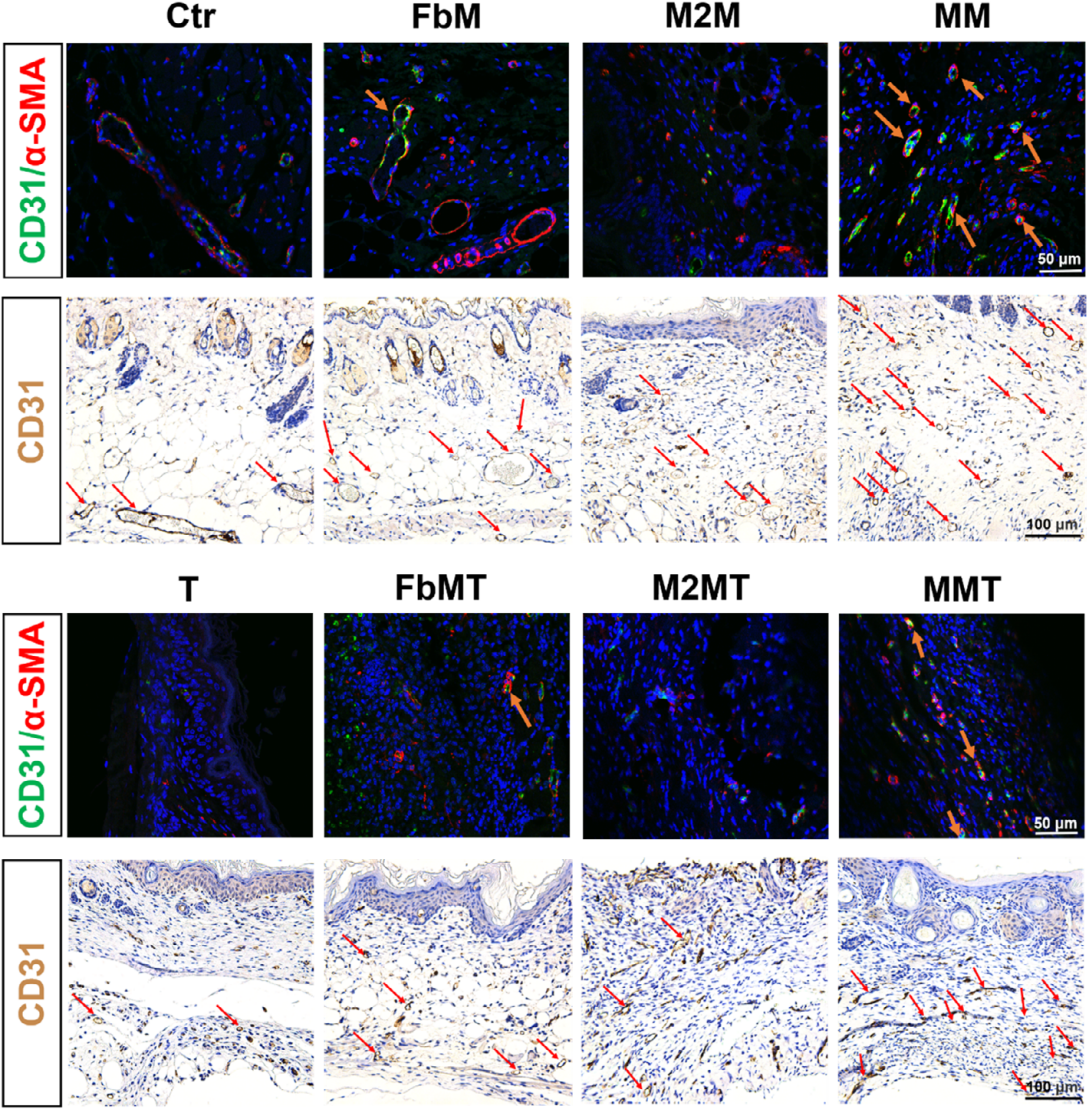
The CD31 immunohistochemical staining and CD31/α‐SMA immunofluorescence staining images of the wounds under different treatments at Day 12

To illustrate the myofibroblast activation and neovascularization, immunohistochemical staining (CD31) and dual immunofluorescence staining (CD31/α‐SMA) were presented in Figure 5. α‐smooth muscle actin (α‐SMA) has been proved to be involved with the conversion of fibroblasts to myofibroblasts,^34^ which further led to migration, proliferation, and production of ECM components. While CD31 was a sensitive and specific marker for vascular differentiation.^35^ Data showed that groups containing fibroblastic membrane fractions (FbM and MM) had stronger effects to promote myofibroblast activation. And also, both the density and number of blood vessels were significantly higher in the MM‐treated group than those in the other groups, referring to a promoted revascularization and matrix reconstruction. In the inhibitor‐treated groups, they only showed small difference from the untreated groups, which certified that the TGF‐β inhibitor would not disturb the dominating effect of cytomembranes.

**FIGURE 6:**
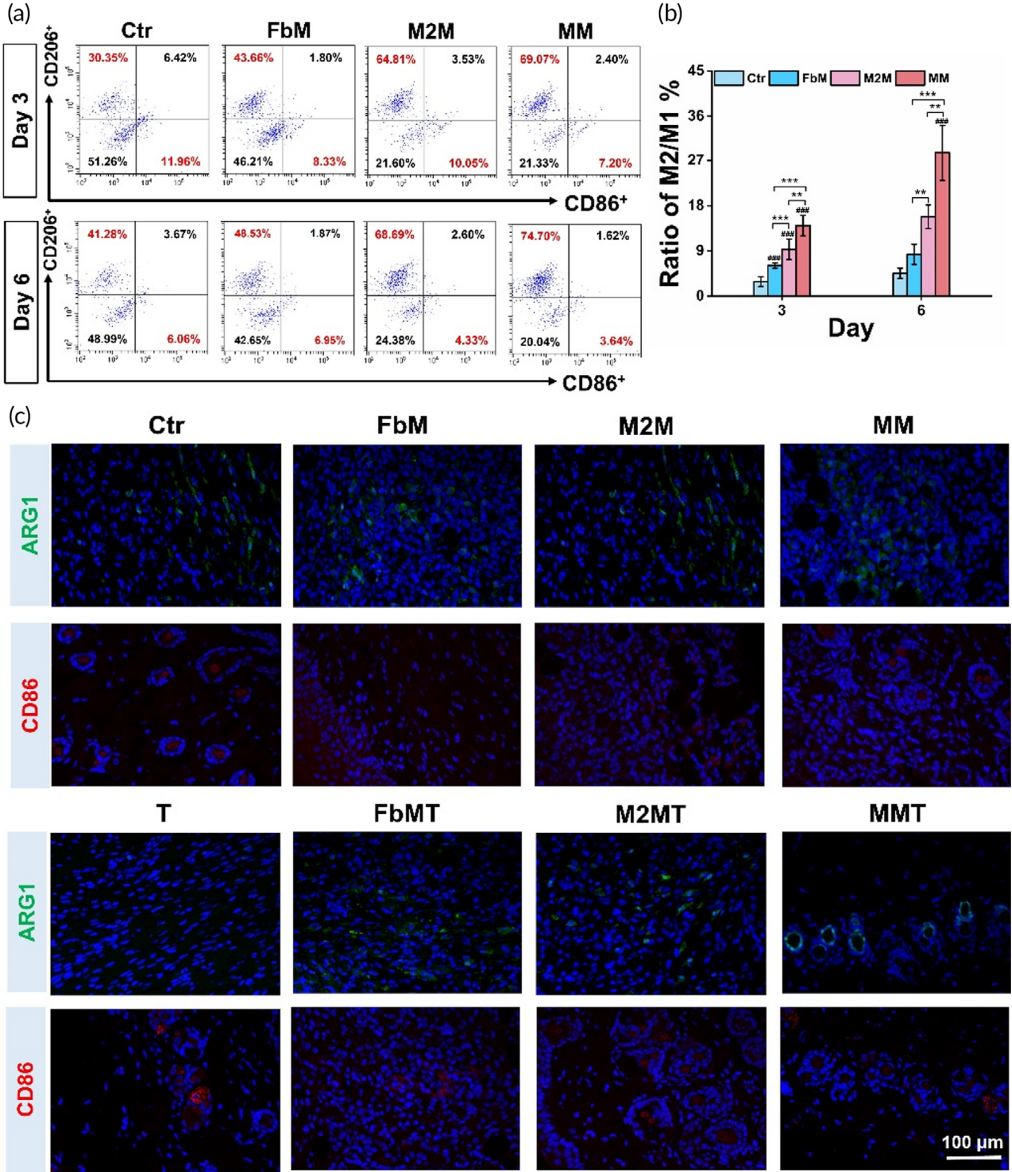
In vivo macrophage infiltration at the wound sites. (a) Flow cytometry determined CD86 (marker of M1 macrophage) and CD206 (marker of M2 macrophage) expression under different treatments for 3 or 6 days and (b) the M2/M1 ratio. (c) Immunofluorescence evaluated ARG1 (marker of M2 macrophage) and CD86 (marker of M1 macrophage) expression for 12 days after membrane treatments. (DAPI: blue, ARG1: green, CD86: red). Data are presented as mean ± SD (*n* = 3). “#” is the intragroup comparison with “Ctr,” and “*” is the intergroup comparison. * or #, *p* < 0.05; ** or ##, *p* < 0.01; *** or ###, *p* < 0.001

Next, to elaborate the in vivo regulatory mechanism of MM, the skin tissues of the wounds were collected and analyzed by flow cytometry assay. In Figure 6a, the M1 macrophages (CD86^+^) were reduced after the membrane treatment for 3 or 6 days, among which the MM showed the strongest effect. It was noted that the FbM also caused the reduction in M1 macrophages, and this phenomenon was consistent with the in vitro results we demonstrated in Figure 2. On the contrary, the M2 macrophages (CD206^+^) were largely increased at Day 3 by all three types of membranes. Although the amount of M2 macrophages was raised in the control group at Day 6 (40.21%), the other three groups further increased the M2 population. As the result, the calculated ratio of M2/M1 in Figure 6b illustrated a highest percentage in MM‐treated group. In the fluorescent images of the wound tissue marked with ARG1 and CD86 (Figures 6c and S11), the M2 macrophage marker (ARG1) was increased in the membrane‐treated groups after wound healing (Day 12), and the intensity of the M1 macrophage marker (CD86) was lower in these groups. These data also validated the repolarization effect of MM on M1 macrophages toward M2 phenotype.

## 3 DISCUSSION

Wound healing is a complex and orderly process including four overlapping phases.^34^ Typical strategies to accelerate full‐thickness wound repair have been focused on the promotion of one specific phase to cause a series of biological responses.^17^, ^35^, ^36^ With the advancement of biomimetic material technology, cytomembranes have been developed to convey more complicated and systematic information due to the abundance of condensed co‐stimulatory molecules.^29^, ^37^ However, few studies have explored whether cytomembrane can modulate the sequential bioactivities in the wound healing process. Previously, as the most important immune and reparative cells in wound repair, macrophages and fibroblasts have been individually manipulated by various biomaterials.^38^, ^39^, ^40^, ^41^ But the concurrent regulation over these two cells are usually failed due to the insufficient signaling molecules in a single material platform.^42^ In this work, we take advantages of the pleiotropic effects carried in cytomembranes and further amplify the synergistic cascade by fabricating mixed cytomembrane biologics derived from parent macrophages and fibroblasts. The resolution of inflammation and promotion of proliferation are simultaneously pursued in our design.

To this end, we first successfully obtained BMDMs‐derived M2 macrophages and skin‐derived fibroblasts (Figure 1) and then extracted their cytomembranes. The primal effects of these two membranes were tested in vitro. As in Figure 2, the re‐education ability of M2 macrophage membrane to reverse pro‐inflammatory M1 macrophages into M2 phenotype was characterized. It was not surprising to see that M2M and MM remodeled macrophages toward M2 phenotype because both of them contained M2 membrane. But it was noted that the fibroblast‐derived FbM also enforced macrophages to demonstrate M2 phenotype. In fact, it had already been reported that the NIH 3T3 fibroblast‐derived conditioned medium caused reduced pro‐inflammatory cytokine production in macrophages,^14^ so the FbM might also limit the excessive generation of pro‐inflammatory cytokines in our study. However, this acute repolarization of macrophages induced by FbM was only observed after 24 h of in vitro treatment, whether it was powerful enough to sustain favorable anti‐inflammation throughout the healing process remained to be further evaluated in the animal study. Meanwhile, we expected the MM to have promotion effect on migration, proliferation, and activation of the endogenous fibroblasts occurred at the wound site. As anticipated, the MM did show promotion effect on all three fibroblastic behaviors (Figure 3), and the individual FbM and M2M also showed positive results, but less strong. As an interstitial cell, fibroblast's behaviors were incorporated in an extracellular matrix comprised of proteins such as collagens and proteoglycans.^1^ Here, the encountering with homologous FbM might strengthen cell–cell junction between fibroblasts, thus enhanced the matrix generation, and promoted the context‐dependent fibroblastic behaviors.^16^ Likewise, the M2M greatly influenced the biological behaviors of fibroblasts as well (Figure 3). This phenomenon was implicated with the M2 macrophage‐derived signals that directly affected the gene expression and protein secretion of fibroblasts,^43^ which were closely correlated with the ECM deposition that changed fibroblasts' behaviors in feedback. Collectively, these results indicated to a well‐inherited nature expressed in the MM from the cytomembranes of both M2 macrophage and fibroblast and was further amplified to exert pleiotropic effects on endogenous macrophages and fibroblasts.

In the preclinical in vivo study, accelerated wound healing was achieved in the MM‐treated mice (Figure 4). To elucidate the roles of the two cytomembranes, we initiated investigation into the changes of macrophages at the wound site. In Figure 5, massive M1 macrophages (CD86^+^) were activated to cause inflammation in the first 3 days after surgery. And the population of M2 macrophages (CD206^+^) in untreated mice was kept low even at Day 6. In accordance with the in vitro results, M2M and MM were highly efficient to reprogram M1 to M2 macrophages. Interestingly, FbM also aroused the macrophages to repolarize in vivo at Day 3, which was consistent with the Western Blot data, we presented in vitro (Figure 2b,c). However, this repolarization was not long lasting under the disturbing in vivo microenvironment at Day 6, suggesting to a relatively poorer effect of FbM in the resolution of inflammation. This phenomenon certified that the particular biofunctions expressed in each cytomembrane were indispensable for the improved wound repair. And this conclusion was further supported by the CD31/α‐SMA staining experiment (Figure 5), in which FbM and MM both achieved preferable results, but M2M was less effective. Until now, by utilizing natural cytomembranes derived from M2 macrophages and fibroblasts, we successfully realized the dual regulation over both the inflammation and proliferation phases. But this expedited development in wound healing might cause another concern, that the overexpression of fibrosis would induce excessive collagen deposition and ECM misalignment, or even dysfunctional and disfiguring scars.^44^, ^45^ Therefore, we intervened the cellular proliferation and ECM production procedures by injecting TGF‐β inhibitor in the late stage of wound healing. Results showed that ARG1 (M2 marker) was reduced, CD86 (M1 marker) was slightly re‐upregulated (Figure 6), and α‐SMA (activated fibroblasts) was decreased (Figure 5). With the interference of TGF‐β inhibitor, the fibrotic overreactions were normalized, entering the advancement toward scarless wound healing.

This study was inspired by our previous work that the hybrid cell membranes from the tumor cells and polarized macrophages can be used as a tumor vaccine.^20^ The regular membrane extraction method, which was presented in this study, mechanically broke the cells and obtained the mixed membranes by centrifugation. The membranous substances were highly efficient to convey desired functions and also came with pleiotropic effects. Here in the skin wound repair process, we further show that simple nature‐derived membranes can modulate complex bioactivities.

## 4 CONCLUSIONS

In this work, a series of injectable biologics based on cytomembranes extracted from M2 macrophages and fibroblasts were prepared to promote full‐thickness wound healing. These cytomembrane biologics demonstrated unique biofunctions like remodeling pro‐inflammatory M1 macrophages to anti‐inflammatory M2 phenotype, and modulating cellular behaviors of fibroblasts. During the wound healing process, macrophages were initially recruited as one of the innate immune cells to activate the inflammatory responses. The cytomembrane biologics reprogrammed M1 macrophages to M2 phenotype, which significantly reduced the excessive inflammation process. In addition, the re‐educated M2 macrophages were also intimately involved in fibroblastic responses and blood vessels regeneration. Meanwhile, the fibroblast membrane contained in the biologics promoted the migrative and proliferative behaviors of the endogenous fibroblasts, forcing them to transform into myofibroblasts. Thus, these aligning cascade led to a more preferable ECM production, as well as angiogenesis and tissue remodeling. By interfering the late stage of wound healing with the TGF‐β inhibitor, the fibrotic overreactions were ceased and the regular collagen arrangement was promoted. Thus, the dual modulation over these two cell types synergistically accelerated the wound healing. This natural membrane‐based biologics might be further developed as an efficient bridging material to enhance the material–bio interactions and inflict pleiotropic effect in regenerative studies.

## 5 MATERIALS AND METHODS

### 5.1 Materials

Lipopolysaccharides (LPSs) were obtained from Sigma Aldrich. Cell counting assay kit (CCK8) was purchased from MCE (China). Phenylmethane‐sulfonyl fluoride (PMSF) and 3, 3′‐dioctadecyloxacarbocyanine perchlorates (DiO) were purchased from Beyotime Institute of Biotechnology (China). Mouse recombinant granulocyte macrophage colony‐stimulating factor (GM‐CSF) and interleukin‐4 (IL‐4) were provided by Peprotech (Rocky Hill, CQ). Red blood cells lysis buffer and the Smart‐ECL system were purchased from Beijing Solarbio Science & Technology Co., Ltd (China). Dulbecco's modified Eagle's medium (DMEM), RPMI 1640 medium, fetal bovine serum (FBS), penicillin–streptomycin, trypsin, and collagenase were obtained from Thermo Fisher Scientific. TGF‐β inhibitor was provided by Selleck. The crystal violet solution was purchased from Sangon Biotech (Shanghai, China). PVDF membranes were purchased from Millipore. Anti‐CD86‐PE (Clone GL1, Cat. No. 553692) and anti‐CD206‐Alexa 647 (Clone MR5D3, Cat. No. 565250) were purchased from BD Biosciences. Anti‐β‐Actin (HC201‐02) was provided by Beijing TransGen Biotech Co., Ltd. (China). Anti‐iNOS (ER1706‐89) was obtained from HUABIO (Hangzhou, China). Anti‐N‐cadherin (ab76011) and anti‐PCNA (ab29) were purchased from Abcam. Anti‐Akt (A5525) and anti‐p‐Akt (Ser473) (A5030) were provided by Bimake (Shanghai, China). Anti‐Vimentin (5741S), anti‐E‐cadherin (3195S), anti‐CD206 (24595T), anti‐ERK1/2 (4695S), anti‐p‐ERK1/2 (4370S), anti‐Snail (3879S) and anti‐α‐Smooth Muscle Actin (19245S) were obtained from Cell Signaling Technology. Anti‐ARG1 (sc‐166920) and anti‐Twist (sc‐81417) were purchased from Santa Cruz Biotechnology. Anti‐CD31/PECAM‐1 (AF3628) was provided by R&D Systems (USA). Anti‐CD86 (BU63) was obtained from Novus Biologicals.

### 5.2 Mice and cell lines

BALB/c mice (female, 5–7 weeks) and KM mice (female, 6–8 weeks) were purchased from Chongqing Medical University (Chongqing, China). The animal study was reviewed and approved by the Institutional Animal Care and Use Committee (IACUC) of Chongqing Medical University, School of Medicine. The NIH 3T3 cells, MEF cells and RAW264.7 macrophages were cultured in DMEM medium containing 10% FBS, 1% penicillin–streptomycin (10,000 U/ml).

### 5.3 Extraction and culture of mouse skin fibroblasts

After removing the back hair as much as possible, the female BALB/c mice were sacrificed and the back skin tissues were harvested. The skin was soaked in PBS containing 6% penicillin–streptomycin for 5 min and washed with PBS for three times. After cutting the tissue into pieces, washed for three times with PBS and centrifuged at 800 g for 5 min to collect the precipitate. Treated by 0.25% trypsin and collagenase IV for 6 h. After filtering with 200 mesh filter, the filtrate was centrifuged at 800 g for 10 min to collect the cells and washed again with PBS for two times. The skin fibroblasts were cultured in 100 mm dishes with DMEM medium containing 20% FBS and 1% penicillin–streptomycin.

### 5.4 Generation of bone marrow‐derived macrophages

The female BALB/c mice were sacrificed, both femur and tibiae were collected, muscle attachment was removed. Intact bones were soaked in PBS containing 10% penicillin–streptomycin for 5 min and washed with PBS for three times. Both bone joint heads were cut off, and the bone cavities were flushed with DMEM medium using a 1 ml syringe. After centrifugation at 400 g for 5 min, the red blood cells (RBCs) were lysed with lysis buffer. The cells were collected and cultured in 100 mm dishes containing DMEM medium added with 20% heat‐inactivated FBS, 1% penicillin–streptomycin; 24 h later, the culture medium was changed with 10% heat‐inactivated FBS, 1% penicillin–streptomycin and GM‐CSF (20 ng/ml). M1 macrophages can be harvested from the LPS (20 ng/ml) treatment, and M2 macrophages can be obtained after treatment with IL‐4 (20 ng/ml). After 1 week of conditioned culturing, these macrophages were harvested for further use.

### 5.5 Preparation of membrane biologics

The M2 macrophages and fibroblasts were mixed at the ratio of 1:1 (cell number) and collected with a cell scraper and washed with PBS for three times. After centrifugation at 400 g for 5 min, the cell pellets were resuspended in hypotonic solution containing 10 mM PMSF. After incubating on ice for 10 min, the solution was ultrasonically broken in ice bath for 6 min and centrifuged at 700 g for 10 min. The supernatant was collected and centrifugated at 14,000 g for 40 min. The precipitate was collected and freeze‐dried, stored at −80°C for further use.

### 5.6 CCK8 assay

The cells were seeded in a 96‐well plate at a density of 5 × 10^3^ cells/well. After being cultured in incubators for 4 h, the cells were incubated with FbM, M2M or MM, respectively. At the time interval of 0, 12, 24, and 48 h, the medium was discarded. The cells were collected and washed with PBS, followed by staining with freshly prepared CCK8 solutions for 2 h at 37°C. The absorbance used to calculate the cell viability was measured 450 nm.

### 5.7 Scratch assay

The cells were planted in six‐well plates. When the confluence reached 80%–90%, a uniform scratch was formed by a sterile pipette tip. The cells were cultured in serum‐free medium as the control group (Ctr), or in the same medium added with FbM, M2M or MM. At 0 and 12 h after the scratch, the cell plates were observed under the bright field of electron microscope. The width of the scratch was measured to calculate the relative closure rate.

### 5.8 Migration assay

The vertical migration of cells was determined in polycarbonate transwell inserts with 8 μm of pore diameter. Under sterile operation, 750 μl of complete medium containing FbM, M2M, or MM was added into the 24‐well plate, the transwell chamber was placed in the well. The 2 × 10^5^ of NIH 3T3 cells were seeded in the upper inserts and cultured for 12 h. Then the cells on the upper surface of the cell filter were removed, the migrated cells on the lower surface were fixed with methanol at room temperature for 20 min. The 0.2% crystal violet solution was used for dyeing at room temperature for 30 min. After that, the chambers were washed twice with PBS. The cells on the lower surface of cell filter were observed and photographed under microscope. The number of migrated cells in each group was counted.

### 5.9 Western blot

The cells were seeded in six‐well plate at the density of 2 × 10^5^ cells/well; 24 h later, FbM, M2M, or MM were added into the medium. After another 24 h, the cells were washed with PBS. The 1% SDS lysis buffer was used to extract protein in samples and the BCA protein quantification kit was used to determine the concentration. A total of 10–20 μg protein was used for electrophoresis. The volume of protein and the corresponding volume of 5× SDS loading buffer were calculated based on the measured concentration. After mixing, the samples were heated in a metal bath at 95°C for 10 min. Sodium dodecyl sulfate‐polyacrylamide gels were prepared in advance, and the target proteins were separated at a constant pressure of 80 V after adding the protein sample. Subsequently, proteins were transferred to PVDF membrane under a constant current of 300 mA. The membranes were blocked in 5% bovine serum albumin at room temperature for 1 h and then incubated with the primary antibody including anti‐β‐Actin (1:5000), anti‐CD206 (1:1000), anti‐iNOS (1:1000), anti‐ARG1 (1:1000), anti‐E‐cadherin (1:1000), anti‐N‐cadherin (1:1000), anti‐Vimentin (1:1000), anti‐PCNA (1:1000), anti‐Snail (1:1000), anti‐Twist (1:1000), anti‐ERK1/2 (1:1000), anti‐P‐ERK1/2 (1:1000), anti‐Akt (1:1000) and anti‐P‐Akt (1:1000) at 4°C overnight. The next day, the membranes were washed for five times with TBST, 5 min for each time, and then combined with the secondary antibody for 1.5 h at room temperature. The results were imaged with a chemiluminescence imager through the Smart‐ECL system.

### 5.10 Immunofluorescence assay

The 1 × 10^4^ cells were seeded in 24‐well plate covered with sterile cell slides, 12 h later, the FbM, M2M or MM were separately added, the cells were cultured for 24 h. The cells were washed with PBS for two times and then fixed by 4% paraformaldehyde at 37°C for 20 min. Subsequently, the cells were washed with PBS for three times, penetrated by PBS containing 0.2% TritonX‐100 for 10 min, and washed again with PBS for three times. The 10% BSA solution was used for blocking at 4°C overnight. The next day, they were incubated with primary antibody (1:100) at 4°C for 12 h. After washing with PBST for four times, the cell slides were stained with DAPI, phalloidin, and fluorescent secondary antibody at 200:1 dilution at room temperature for 90 min. After the samples were processed by the fluorescence quencher, the images of cells were taken by the confocal laser scanning microscope.

### 5.11 Animal model and surgery

The BALB/c mice and KM mice were used to build the animal model of skin repair. The mice were randomly divided into eight groups named as Ctr (untreated group), FbM (fibroblast membrane‐treated group), M2M (M2 macrophage membrane‐treated group), MM (mixed membrane‐treated group), T (TGF‐β‐treated group), FbMT (fibroblast membrane and TGF‐β‐treated group), M2MT (M2 macrophage membrane and TGF‐β‐treated group), and MMT (mixed membrane and TGF‐β‐treated group). A skin wound with the diameter of 8 ± 0.5 mm on the back was caused using a hole punch tool. The cytomembrane biologics were injected around the wound at the dosage of 2 μg/mouse. After the surgery, the size and photographs of the wounds were recorded every other day. At 7 days, the T, FbMT, M2MT, and MMT groups were injected with the TGF‐β inhibitor around the skin defect. The skin tissues of the wound were harvested at 12 days and then immersed in 4% paraformaldehyde to prepare tissue sections for histological analysis (H&E, Masson, CD31, and CD31/α‐SMA immunofluorescence). For the analysis of macrophage polarization, the tissues were cut into pieces and digested with trypsin and collagenase IV, and the cells were labeled with anti‐CD45‐APC‐Cy7, anti‐CD11b‐FITC, anti‐F4/80‐BV421TM, anti‐CD86‐PE, and anti‐CD206‐Alexa 647 antibodies for flow cytometry analysis.

### 5.12 Animal imaging assay

The animal imaging assay was used to observe the degradation of membrane in the wound. The extracted cell membranes were prestained with DiO. The BALB/c mice were randomly divided into PBS group and M@DiO group. After injecting the membrane solution into the edge of wounds, the degradation pattern of cytomembrane was recorded by the animal imaging system (Lumina XR) at Days 2 and 4 after the surgery.

### 5.13 Statistical analysis

All data were expressed as mean ± standard deviations (SD). Student's *t*‐test was used to statistically analyze the data obtained from the experiments. “#” is the intragroup comparison with “Ctr,” and “*” is the intergroup comparison. Significant differences are indicated as * or #, *p* < 0.05; ** or ##, *p* < 0.01; *** or ###, *p* < 0.001.

## AUTHOR CONTRIBUTIONS

**Dongqing Wang:** Investigation (lead); methodology (lead); writing – original draft (equal). **Heying Chen:** Data curation (lead); investigation (equal); methodology (equal). **Li Lei:** Data curation (equal); investigation (equal); methodology (equal). **Jun Chen:** Data curation (equal); validation (equal). **Jimin Gao:** Conceptualization (equal); formal analysis (equal). **Jiahe Liu:** Data curation (equal); methodology (equal). **Qianyin Li:** Project administration (equal). **Yajun Xie:** Formal analysis (equal). **Yilu Ni:** Funding acquisition (lead); project administration (lead); writing – review and editing (equal). **Yi Hu:** Supervision (lead); funding acquisition (equal); validation (equal).

## CONFLICT OF INTEREST

The authors have declared that no competing interest exists.

### PEER REVIEW

The peer review history for this article is available at https://publons.com/publon/10.1002/btm2.10344.

## Supporting information


**Figure S1** The relative cell viability of the MEF cells under different treatments. “Ctr” represents untreated group. Data are presented as mean ± SD (*n* = 3). “#” is the intra‐group comparison with “Ctr”, and “*” is the inter‐group comparison. * or #, *P* < 0.05; ** or ##, *P* < 0.01; *** or ###, *P* < 0.001.6l.
**Figure S2**. The relative cell viability of the skin‐derived fibroblasts under different treatments. “Ctr” represents untreated cells. Data are presented as mean ± SD (*n* = 3). “#” is the intra‐group comparison with “Ctr”, and “*” is the inter‐group comparison. * or #, *P* < 0.05; ** or ##, *P* < 0.01; *** or ###, *P* < 0.001.6l.
**Figure S3**. The expression of N‐cadherin, E‐cadherin, Vimentin, PCNA, Snail and Twist in mice‐derived fibroblasts under different treatments for 24 h.6l.
**Figure S4**. Akt, Erk1/2 expression, and their phosphorylation levels in mice‐derived fibroblasts under different treatments for 24 h.6l.
**Figure S5**. In vivo degradation of the DiO‐stained MM cytomembranes.6l.
**Figure S6**. In vivo evaluation of wound healing in KM mice.6l.
**Figure S7**. Quantitative evaluation of the wound closure rate in KM mice.6l.
**Figure S8**. The H&E staining images of the wound area in KM mice.6l.
**Figure S9**. Analysis of the M2/M1 ratio by flow cytometry in KM mice. * or #, *P* < 0.05; ** or ##, *P* < 0.01; *** or ###, *P* < 0.001.6l.
**Figure S10**. Corresponding data calculated from Figure 4f.6l.
**Figure S11**. Corresponding data calculated from Figure 6c.

## Data Availability

The data that supports the findings of this study are available in the supplementary material of this article.
