# Supplementary material for: Biofabricated macrophage and fibroblast membranes synergistically promote skin wound healing
Source: Bioeng Transl Med. 2022 Jun 3;7(3):e10344. doi: 10.1002/btm2.10344 (PMC9472019; doi:10.1002/btm2.10344)
Supplement: Supplementary file 1 — Figure S1 The relative cell viability of the MEF cells under different treatments. “Ctr” represents untreated group. Data are presented as mean ± SD (n = 3). “#” is the intra‐group comparison with “Ctr”, and “*” is the inter‐group comparison. * or #, P < 0.05; ** or ##, P < 0.01; *** or ###, P < 0.001.6l. Figure S2. The relative cell viability of the skin‐derived fibroblasts under different treatments. “Ctr” represents untreated cells. Data are presented as mean ± SD (n = 3). “#” is the intra‐group comparison with “Ctr”, and “*” is the inter‐group comparison. * or #, P < 0.05; ** or ##, P < 0.01; *** or ###, P < 0.001.6l. Figure S3. The expression of N‐cadherin, E‐cadherin, Vimentin, PCNA, Snail and Twist in mice‐derived fibroblasts under different treatments for 24 h.6l. Figure S4. Akt, Erk1/2 expression, and their phosphorylation levels in mice‐derived fibroblasts under different treatments for 24 h.6l. Figure S5. In vivo degradation of the DiO‐stained MM cytomembranes.6l. Figure S6. In vivo evaluation of wound healing in KM mice.6l. Figure S7. Quantitative evaluation of the wound closure rate in KM mice.6l. Figure S8. The H&E staining images of the wound area in KM mice.6l. Figure S9. Analysis of the M2/M1 ratio by flow cytometry in KM mice. * or #, P < 0.05; ** or ##, P < 0.01; *** or ###, P < 0.001.6l. Figure S10. Corresponding data calculated from Figure 4f.6l. Figure S11. Corresponding data calculated from Figure 6c. [file BTM2-7-e10344-s001.docx]

**Biofabricated macrophage and fibroblast membranes synergistically promote skin wound healing**

Dongqing Wang^1, #^, Heying Chen^1, #^, Li Lei^1, #^, Jun Chen^2^, Jimin Gao^3^, Jiahe Liu^1^, Qianyin Li^1^, Yajun Xie^1^, Yi Hu^2, *^, Yilu Ni^1, **^

^1^The M.O.E. Key Laboratory of Laboratory Medical Diagnostics, The College of Laboratory Medicine, Chongqing Medical University, #1 Yixueyuan Road, Yuzhong District, Chongqing, 400016, China

^2^CAS Key Laboratory for Biomedical Effects of Nanomaterials and Nanosafety, Institute of High Energy Physics and University of Chinese Academy of Sciences (UCAS), Chinese Academy of Sciences (CAS), Beijing, 100049, China

^3^Zhejiang Provincial Key Laboratory for Technology & Application of Model Organisms, School of Laboratory Medicine and Life Science, Wenzhou Medical University, University Town, Wenzhou, Zhejiang 325035, China

^#^These three authors contributed equally to this work.

**Correspondence: Yilu Ni, Chongqing Medical University, #1 Yixueyuan Road, Yuzhong District, Chongqing, 400016, China. E-mail: nyl@cqmu.edu.cn

^*^Correspondence: Yi Hu, Institute of High Energy Physics and University of Chinese Academy of Sciences (UCAS), Chinese Academy of Sciences (CAS), Beijing, 100049, China. E-mail: huyi@ihep.ac.cn

**Supplementary Figures**

**

Figure S1**. The relative cell viability of the MEF cells under different treatments. "Ctr" represents untreated group. Data are presented as mean ± SD (*n* = 3). "#" is the intra-group comparison with "Ctr", and "*" is the inter-group comparison. * or #, *P* < 0.05; ** or ##, *P* < 0.01; *** or ###, *P* < 0.001.

**

Figure S2**. The relative cell viability of the skin-derived fibroblasts under different treatments. "Ctr" represents untreated cells. Data are presented as mean ± SD (*n* = 3). "#" is the intra-group comparison with "Ctr", and "*" is the inter-group comparison. * or #, *P* < 0.05; ** or ##, *P* < 0.01; *** or ###, *P* < 0.001.


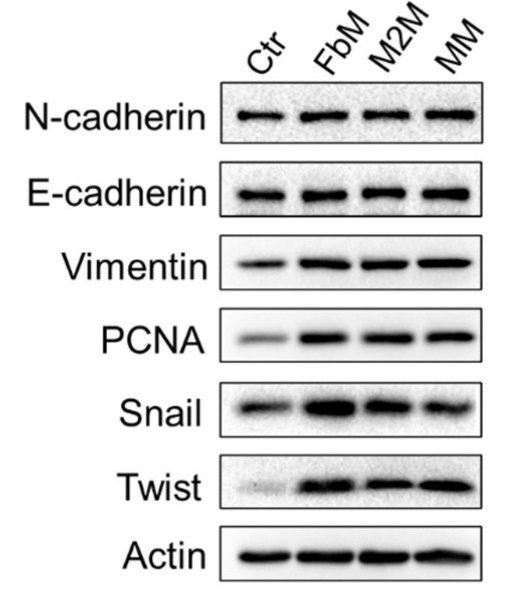
**Figure S3**. The expression of N-cadherin, E-cadherin, Vimentin, PCNA, Snail and Twist in mice-derived fibroblasts under different treatments for 24 h.


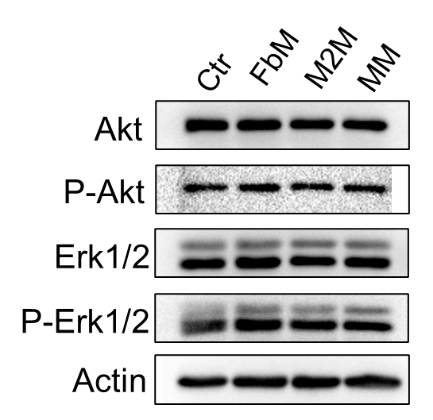
**Figure S4**. Akt, Erk1/2 expression, and their phosphorylation levels in mice-derived fibroblasts under different treatments for 24 h.


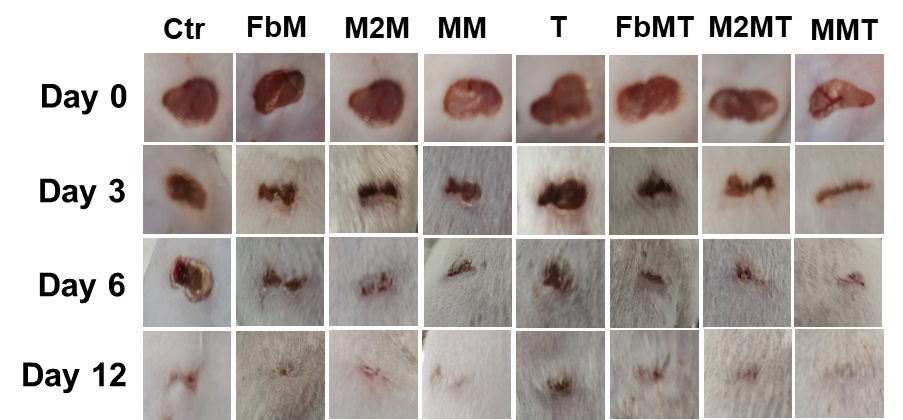

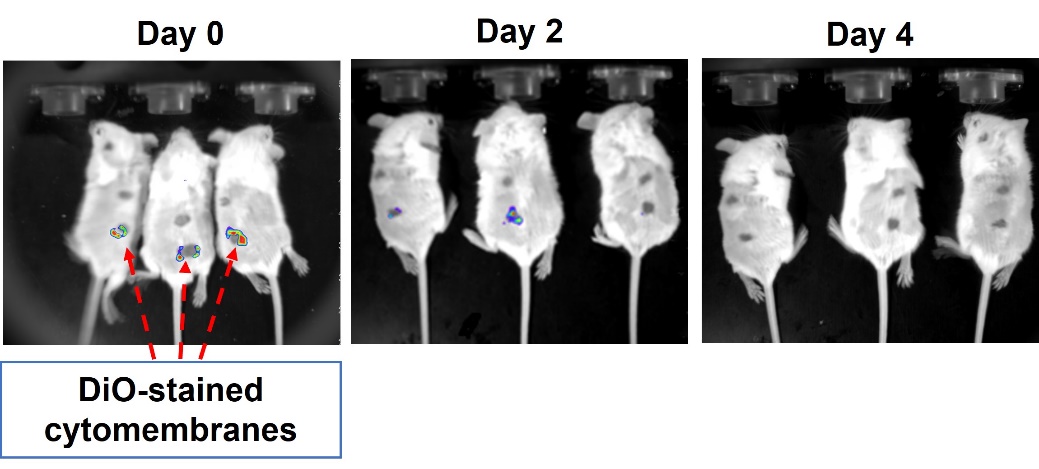
**Figure S5**. *In vivo* degradation of the DiO-stained MM cytomembranes.



**Figure S6**. *In vivo* evaluation of wound healing in KM mice.

**Figure S7**. Quantitative evaluation of the wound closure rate in KM mice.


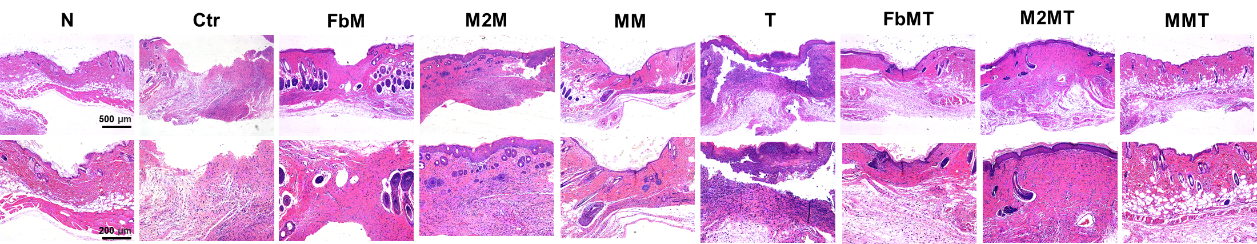
**Figure S8**. The H&E staining images of the wound area in KM mice.



**Figure S9**. Analysis of the M2/M1 ratio by flow cytometry in KM mice. * or #, *P* < 0.05; ** or ##, *P* < 0.01; *** or ###, *P* < 0.001.

**

Figure S10**. Corresponding data calculated from Figure 4F.


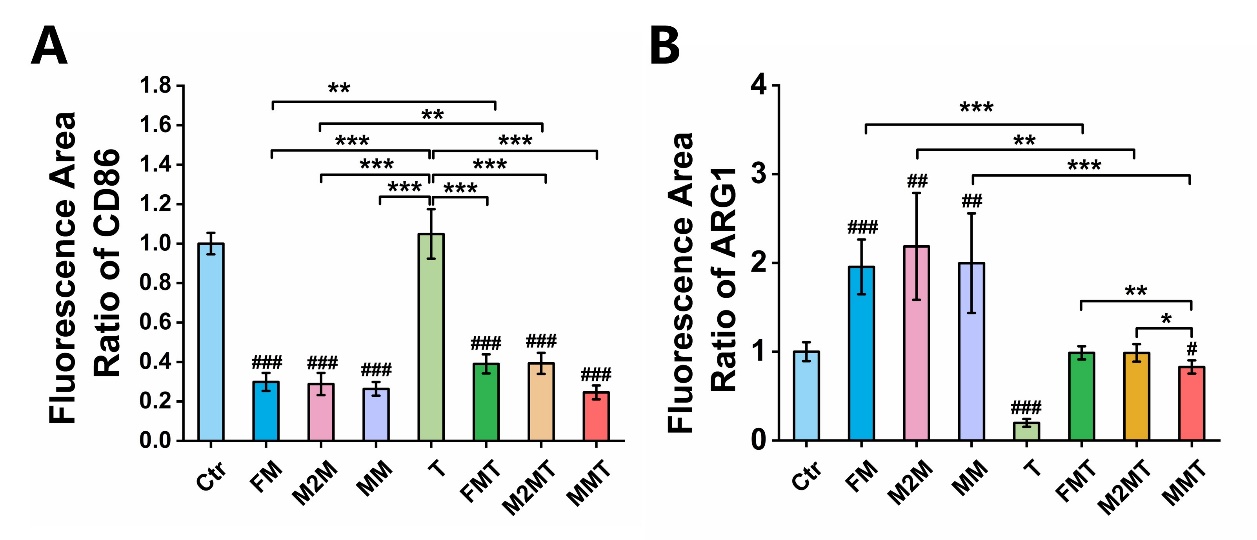
**Figure S11**. Corresponding data calculated from Figure 6C.
